# Supplementary material for: A UMLS-based spell checker for natural language processing in vaccine safety
Source: BMC Med Inform Decis Mak. 2007 Feb 12;7:3. doi: 10.1186/1472-6947-7-3 (PMC1805499; doi:10.1186/1472-6947-7-3)
Supplement: Additional file 13 — RAP application directory. Sets up directory for RAP (RDF application for PHP) source code [file 1472-6947-7-3-S13.gz › rap/doc/phpdoc/li_resModel.html]

xml version="1.0" encoding="iso-8859-1"?


resModel

Description
:   Class trees  
    Index of elements  
    Todo List

Classes
:   ResAlt
:   ResBag
:   ResContainer
:   ResIterator
:   ResList
:   ResLiteral
:   ResModel
:   ResProperty
:   ResResource
:   ResSeq

Files
:   ResAlt.php
:   ResBag.php
:   ResContainer.php
:   ResIterator.php
:   ResList.php
:   ResLiteral.php
:   ResModel.php
:   ResProperty.php
:   ResResource.php
:   ResSeq.php

phpDocumentor v 1.3.0RC3
